# Mind the gender gap: The social neuroscience of belonging

**DOI:** 10.3389/fnhum.2023.1094830

**Published:** 2023-04-05

**Authors:** Gina Rippon

**Affiliations:** College of Health and Life Sciences, Institute of Health and Neurodevelopment, Aston University, Birmingham, United Kingdom

**Keywords:** social brain, gender gap, inclusion, belonging, social rejection, expectancy-value, predictive coding, stereotypes

## Abstract

Gender gaps persist in the 21st century, in many aspects of society and in many types of organisation. There are earnings gaps in almost all domains, reports of glass ceilings and the “missing middle” in business, finance, law and politics, and dramatic under-representation of women in many branches of science, even in the most “gender equal” countries. This is despite decades of effort to address them, including targeted legislation and many Diversity and Inclusion initiatives. Early essentialist, competence-based explanations for the existence of gender gaps have been largely discredited at the research level, although their persistence in the public consciousness and at the level of education and training can still negatively bias both individual self-belief and organisational processes. Contemporary essentialist explanations are now emerging, with claims that such gaps are the manifestations of the presence or absence of endogenous, brain-based characteristics underpinning career progression or career preferences. The focus remains on the individual as the source of gender imbalances. Less attention has been paid to the contextual aspects of organisations where gender gaps are evident, to inclusion (or the lack of it), or the availability of unbiased reward and progression pathways. Advances in 21st century social cognitive neuroscience are revealing the importance of external organisational processes as powerful brain-changing forces, with their potentially negative impact on self-belief and a sense of belonging. Key research is demonstrating the cortical and behavioural consequences of negative social experiences, with the activation of core inhibitory pathways associated with low self-esteem, lack of engagement, and eventual withdrawal. This paper will argue that reference to such research will provide better explanations for the persistence of gender gaps, and offer evidence-based insights into addressing gender gap issues. Importantly, this is not a rejection of an endogenous, brain-based explanation for gender gaps but the elaboration of a better-informed 21st century model, flagging up the need to take factors such as cultural stereotyping and organisational bias into account in any drive toward true gender equity, or genuinely levelled playing fields.

## Introduction

For the last 16 years, the World Economic Forum has produced an annual Global Gender Gap Index report on their measures of gender equality in over one hundred different countries. In 2022, the report showed that, at the current rate of progress, it will take over 130 years to close the global gender gap; this is up from 99 years in 2019, more than a generation later than the previous estimate.^1^ Although this will invariably reflect pandemic effects, the fact that women appear to have been differentially affected should be noted. In the UK, only 1 in 25 of Chief Executive Officers (CEOs) in the largest publicly listed companies are women^2^; in the US, only 6.6% of women hold CEO positions in the S&P 500 list.^3^ In science and technology, the picture is similarly poor; Engineering UK reports that 16.5% of all engineers are women^4^; European data show that only 17% of ICT specialists are women.^5^ Globally, with respect to key future technologies, the proportion of women in the workforce is low: Data and AI (25%), Engineering (15%) Cloud Computing (12%).^6^

Research has shown that more diverse teams can be more creative and productive (Gomez and Bernet, 2019; Tang, 2019). In science, technological innovations mean that the world needs many more specialists in the STEM (Science, Technology, Engineering, and Maths) subjects than it is currently training. A report from the UK’s Royal Society in 2019 showed that the demand for data scientists has increased by nearly 300% over the last 5 years,^7^ the United States has identified a need to increase the number of STEM graduates by 34% to meet forecast demands.^8^ So understanding the origins of gender gaps will be key to overcoming issues associated with lack of diversity or overall lack of engagement with key areas of development.

Early research approaches focussed on the individual, either in terms of biologically-determined competencies or in terms of socially-determined role expectations. Shortcomings in both approaches have become obvious, although the consequent gender stereotypes are still evident as powerful gatekeepers in entry to key areas (Cheryan et al., 2015).

## Gender gaps and the brain–Deficit/difference models

The emergence of brain science at the end of the 18th century heralded a marked “hunt the difference” agenda’ with respect to female and male brains and behaviour, accompanied by the development of many and varied proxy measures for brain structure and function (Rippon, 2019). Although it was in some cases acknowledged that most measures indicated a degree of overlap between data from females and males, the overall impression was generated that were two distinct categories, a female brain linked to feminine characteristics and a male brain linked to masculine characteristics.

This essentialist doctrine claimed that the origins and outcomes of predetermined biological programmes were fixed or “hard-wired,” inevitable and invariant. Early essentialist explanations for gender gaps focused almost exclusively on a brain-based deficit model, with women’s inferior position in society, and absence from positions of power, explained by the inferiority of their intellect, causally linked to the inferiority of their brains (Le Bon, 1879). This was later replaced by an evolutionarily informed “complementarity” model, with female and male brains underpinning different and complementary portfolios of skills and temperaments (Schiebinger, 1991). Early references to the balance between intellectual superiority (necessary for male breadwinners) and moral superiority (necessary for wives and mothers) to ensure “the early education of our children, not to mention the happiness of our homes” (Darwin, 1888) were subsequently replaced by reference to the distinction between male information-processing capacities, such as systemising, and female temperamental characteristics such as empathising (Baron-Cohen, 2004).

However, in the last few years, both technological and theoretical advances in cognitive neuroscience, as well as critical scrutiny of early research interpretations, have revealed fundamental flaws in the assumptions behind the binary categorisation of brains into female or male, and of cognitive skillsets and temperamental characteristics as distinctively feminine or masculine (Hyde et al., 2019). A key finding in 2021 was a comprehensive synthesis of human brain studies which revealed minimal, if any, female/male differences in brain structural characteristics. A similar survey of task-based functional Magnetic Resonance Imaging findings failed to find any reproducible brain activation differences between women and men in verbal, spatial or emotional processing, all spheres which have been invoked in explanations of gender gaps. The author of the survey has urged us to “Dump the Dimorphism” in the light of the lack of any meaningful female/male brain differences revealed to date (Eliot et al., 2021).

With respect to findings in the realms of experimental psychology, similarly historically focussed on measuring differences between feminine and masculine characteristics, analogous syntheses of relevant studies have demonstrated that such differences have also been significantly overstated, have not been replicated and mainly serve to demonstrate the huge areas of overlap between the data from females and that from males. With respect to core cognitive skills or temperamental characteristics, women and men are more similar than they are different (Hyde, 2014). An additional survey investigated a collection of 106 similar meta-analyses and reported average female-male differences as being small or very small (Zell et al., 2015).

It should be noted that despite the evidence-based challenges outlined above, stereotypical beliefs in female-male brain-based skill differences have proved remarkably difficult to dispel (Rippon, 2019). The idea of innate, fixed, sex-based differences remains well-entrenched in public consciousness, and in applied fields such as education and business.^9^ (Skewes et al., 2018). This could be regretfully dismissed as the failure of practical applications to keep up with theoretical origins. However, such stereotypes can serve both as self-fulfilling prophecies and affect the development of self-efficacy (Shin et al., 2019), and can, as we shall see below, “contaminate” the workplace environment (Aycock et al., 2019; Van Veelen et al., 2019).

Traditional essentialist explanations for gender gaps have focussed on internal, intra-individual, competence-based differences, as the source of imbalances in career and life-style choices and/or achievements. So the failure to find a reliable and consistent evidence base for such assertions has, to date, limited potential neuroscience contributions to solving the existence of gender gaps. Either neuroscience has been unable to find the proof that the brains and behaviour of women and men are different and hence solve the problem of gender gaps, or neuroscience should be looking for other brain-based explanations.

## Gender gaps and social processes–Social identity and the power of stereotypes

Early social psychology models similarly focussed on the individual in the generation of gender gaps. In some approaches, there was a focus on evolutionary forces as determining suitable channelling into gender-appropriate roles (Baron-Cohen, 2004), sustained by unchallenged stereotypical beliefs about the “natural” gendered division of labour and ability (Eagly and Wood, 2012; Master et al., 2021).

Beyond the role of individual differences or deficits, real or perceived, it was clear that interaction with social and cultural expectations, in some cases stereotypical, exerted powerful influences, with a match or mismatch between an individual’s self-identity and social role requirements a determining factor in human life-style choices. Social learning theory specified the processes *via* which such gendered norms and expectations were acquired, to inform gender identity and subsequent gendered preferences and choices (e.g., Martin et al., 2002; Shutts et al., 2013). Personal satisfaction and self-esteem, physical and mental wellbeing, and social cohesion and stability were linked to an appropriate “fit” or congruity between an individual’s gender and the role they assume and/or are expected to assume in society. Role incongruity, or gender role violation, was associated with negative outcomes such as poor job satisfaction, lack of commitment and disengagement (Eagly and Diekman, 2004; Diekman et al., 2010). Thus, individual compliance with established gendered expectations, even if inaccurate and stereotypical, served to perpetuate those very expectations (Eagly and Koenig, 2021).

Studies of “social identity threat” also demonstrated the power of social stereotypes to affect behaviour. This relates to an individual’s concerns that their social category is negatively viewed by others (Tajfel and Turner, 1986; Brown, 2020) and that this could put them at risk for exclusion or biased treatment. This has been shown to affect performance when the negative stereotype comprises an expectation that members of certain groups will fail or underperform either at a specific task or demonstrate some kind of overall deficit (Hall et al., 2015).

Initial social constructionist approaches to the problem of gender gaps focussed on the concept of an individual’s social behaviour (including, for example, career choices) being determined by social programming. Unlike the early biological determinist views, this offered the possibility that change could be effected and gender gaps reduced. Reducing or removing stereotypical expectations should remove the power of social pressures to shape individual behaviour. Yet decades of Diversity and Inclusion initiatives did not appear to have significantly increased the rate of progress toward true gender equity or dramatically reduced gender gaps. As with the biological determinist approach, more sophisticated explanations appear to be required.

## Advances in social cognitive neuroscience

### The socially embedded brain

As we have seen, traditional neuroscience explanations for gender gaps in society, framed in terms of fixed, binary sex differences in the brain causally linked to fixed, binary sex differences in cognitive skills sets and/or temperamental characteristics, have not stood the test of time. The individual competence-based contributions of neuroscience to social problems such as gender gaps have turned out to be inaccurate and inadequate, lacking in explanatory utility.

However, contemporary views of how the brain interacts with the outside world potentially offer models more useful to organisational issues. The focus here is not just on individual-based cognitive competence but on the role of brain processes in ensuring that individuals are socially secure and well embedded in any relevant social networks. This social cognitive neuroscience approach emphasises that the brain does not just provide the fundamental bases of human abilities such as language, abstract thought and artistic and scientific creativity. It also ensures our survival and success as social beings, as individuals who *need* to interact with others, and whose behaviour will constantly be adapted to fulfil that need (Dunbar and Shultz, 2007; Lieberman, 2013).

Within this area of social cognitive neuroscience, an emerging consensus has identified a network of key structures that underpin human social behaviour. Of relevance to our understanding of those social processes that might underpin career choice and gender gaps, research has explored the cortical responses to negative social experiences, or “social pain” (Eisenberger, 2012, 2015; Tomova et al., 2021).

A range of online, scanner-based tasks have been developed to model experiences of social rejection, including apparent exclusion from an online team game (Williams and Jarvis, 2006), or “eavesdropping” on negative personal assessments (Eisenberger et al., 2011; Dalgleish et al., 2017). Brain activation has been identified in the bilateral medial prefrontal cortex, an area associated with self-identity and social evaluation (Amodio and Frith, 2006; Lieberman et al., 2019). The bilateral insula and anterior cingulate cortex (ACC) are also involved. These areas are linked to the processing of aversive states such as physical pain (Lieberman and Eisenberger, 2015) or with social pain (Somerville et al., 2006). The ACC is sometimes dubbed the GoNoGo or the “error-evaluation” area, as it is consistently activated in tasks requiring inhibition, either to withhold a response or to change a response following an error (Braver et al., 2001; Steele et al., 2014).

This social pain approach focuses on the loss of self-esteem associated with negative social experiences, which activates brain areas underpinning avoidance behaviour, inhibition and withdrawal (Lieberman, 2013). Pathologically, activation of this network is shown in clinical depression, often linked to severe behavioural inhibition and withdrawal (Porcelli et al., 2019). So the experience or anticipation of social rejection, of not belonging, is linked to powerful inhibitory forces in the brain and to behavioural avoidance and withdrawal.

Individual differences in sensitivity to rejection confirmed the network of cortical areas involved–ACC, medial prefrontal cortex–(Kross et al., 2007), with variations in psychometrically-measured rejection sensitivity (RS) linked to different levels of activation in frontal areas associated with cognitive control. Low RS individuals showed greater activity in these areas as well as reporting lower levels of distress, interpreted as showing greater regulatory activity in situations associated with rejection. It is interesting to note that a high RS-type response has been shown in adolescents during social evaluation tasks, with diminished regulatory responses and more powerful recruitment of socio-affective brain circuitry (Somerville, 2013). It is, of course, at this age that many career-based decisions which may ultimately contribute to gender gaps occur.

There have been neuroscientific studies of the kind of processes identified within social psychology as key to organisational choice and engagement, such as threats to social identity. Studies of the behavioural correlates of the very task-specific, performance-related process dubbed “stereotype threat” have received criticism due to methodological issues and over-interpretation (Stoet and Geary, 2012; but see Spencer et al., 2016). However, cognitive neuroscience research has demonstrated differential patterns of cortical activation using typical stereotype threat paradigms, such as women’s maths performance (Krendl et al., 2008) or spatial cognition ability (Wraga et al., 2007). Studies such as these have identified increased activation in networks, centred around the ACC, associated with emotional self-regulation and the processing of social feedback, rather than in those areas which would normally have been recruited by task demands (Derks et al., 2008).

It would appear that there is an anticipatory component to social pain as well as more fundamental reactive processes– the violation of expectations can activate social pain mechanisms. Chang and Sanfey (2013), using a standard online economic decision-making game, showed that violations of social expectations, as to whether other players were perceived as likely to be fair or unfair, activated the dorsolateral prefrontal cortex and the ACC-insula axis. Both the processing of reward and its anticipation have been shown to be encoded by the anterior cingulate and the striatum (Vassena et al., 2014).

### Predictive coding and the social embedded brain

This future-focussed element links well with another innovative neuroscience concept that could offer insight into human decision-making processes. The predictive coding model of the brain proposes that the brain is not just a hugely efficient, but passive, information processing system, but is a proactive, future-focussed, rule-sampling and rule-generating system. It will generate forward-looking predictions about incoming information, anticipating outcomes, subsequently matching the outcomes to the reality and flagging up any kind of mismatch as a “prediction error” (Friston, 2005, 2009; Clark, 2013). The implications of this view of the brain is the need to appreciate the role of external, rule-based events as data input into the cortical system – the predictions a brain makes, or the decisions it “drives,” will be based on the regularity or predictability of previously encountered environments. So these will need to be scrutinised in order to understand the cortical correlates of typical or atypical behaviour.

The initial focus in predictive coding research was on sensory processing, but it is increasingly being applied to higher level social information, such as predictions linked to social norms or choices of context appropriate behaviour (Chang and Sanfey, 2013; Dunne and O’Doherty, 2013). Similarly, the possession of a “theory of mind” or the ability to predict the knowledge and beliefs of others has been framed as a “neural prediction problem” (Koster-Hale and Saxe, 2013). Difficulties with social processing have been linked to aberrant predictive coding (Kessler et al., 2016).

21st century cognitive neuroscience offers a focus on social acceptance as a fundamental motivational drive in human behaviour (Eisenberger and Lieberman, 2004; Lieberman, 2013; Eisenberger, 2015). Combined with predictive coding models of fundamental brain function, the discipline is now well placed to offer highly relevant brain-based models of the behavioural effects of both the experience of and anticipation of social rejection and exclusion. This could reinvigorate the role of neuroscience in organisational research. A key issue is whether relevant frameworks exist within organisational psychology to utilise these new approaches.

## Social psychology and gender gaps: Belongingness–An expectancy-value approach

Early social constructionist explanations of gender gaps suggested a straightforward link between gendered societal norms and individual life-style choices. For example, the consequences of gender role violation would constitute a negative social experience and avoidance of such would ensure individual compliance and the perpetuation of existing gender gaps.

More nuanced models began to incorporate the concept of a dynamic and interactive interchange between external contextual factors and individual choices and preferences, paying particular attention to pragmatic aspects such as the anticipated social and economic values of life-style decisions. This included a more future-focussed, expectancy feature, where a match between competence-based expectations of success and achievement values, and long-term life goals would be sought. Values were frequently phrased in pragmatic terms, such as income or social standing.

Eccles’ expectancy-value theory has long served as a comprehensive framework for linking such psychological and contextual factors (Eccles, 1987, 1994; Wang and Degol, 2013; Eccles and Wang, 2016). This theory offers valuable insights into the dynamic entanglement of internalised personal factors with external social and cultural influences. A key component of the application of expectancy-value theory to explanations of gender gaps is the matching of perceived competence to anticipated psychological, economic and social costs and benefits of, say, occupational choices. Individual self-beliefs and expectations of success will be entangled with drives to identify the potential values resulting from any given choice, either personal (such as enjoyment or interest) or economic (such as income or monetary benefits) or social (such as reputation or social standing) (Wang and Degol, 2013).

More recently, the focus in the study of social motivation and social decision-making has shifted to more abstract inter-individual social processes, such as the power of group identity and the role of a fundamental motivational drive to identify and belong to congruent and receptive ingroups. This has been termed a sense of belonging or acceptance within a group, or “belongingness.” First outlined by Baumeister and Leary (1995), it emphasised the importance of being involved with others as a universal and fundamental human need. It is linked to the concept of personal involvement in a system or environment as determining behaviour (Anant, 1967), with individuals needing to feel a sense of connectedness with social groups or organisations. It has been defined as “an experience of personal involvement in a system or environment, making people feel to be an integral part of that system or environment” (Hagerty et al., 1992).

Belongingness offers a wider definition of value as a factor in career-based decision-making (Allen et al., 2022). It acknowledges that conscious or unconscious assessment of any expected value may not just be based on potential gains in earnings and/or social status or for altruistic motives, but will also include the perceived opportunity for ingroup membership or inter-personal attachments (Tellhed et al., 2017). The concept of belonging has been widely used in educational and organisational research, and has provided especially fruitful insights into gender gaps within and between different types of organisations and into gender segregated career choice (Malone et al., 2012; Jena and Pradhan, 2018; Master and Meltzoff, 2020).

This has led to a focus on workplace culture and the presence of clues as to the potential for acceptance and inclusion–or clues as to the potential for rejection and ostracism. These can range from straightforward numerical imbalances within an organisation, both vertical and horizontal (Haveman and Beresford, 2012; Van Veelen et al., 2019), to environmental clues reflecting specific stereotypes, such as the equation of computer science with male-only interests (Cheryan et al., 2015), or HR processes or reward systems reflecting a “default male” bias (Stamarski and Son Hing, 2015; Cheryan and Markus, 2020).

Such studies demonstrate that these factors in organisational cultures can have a powerful negative impact on engagement and retention. For example, Van Veelen et al. (2019) carried out an online field survey of female and male STEM workers in the Netherlands. Their report was tellingly entitled “Double Trouble: How Being Outnumbered and Negatively Stereotyped threatens Career Outcomes of Women in STEM.” They measured gender identity threat, or awareness of gender-based negative stereotypes *via* ratings of agreements with statements such as “Sometimes I am concerned that being a woman/man influences how others see me professionally” or “It worries me sometimes that others might judge my work on the basis of my gender.” This measure was then compared to others such as career confidence (“I have a clear sense of what I want to achieve in my career”) and work engagement (“When I get up in the morning, I feel like going to work”). Among women, gender identity threat was predictably greatest among those who worked in organisations with the greatest gender imbalance; and gender identity threat negatively predicted work engagement and career confidence. The story was different for men; any evidence of gender identity threat was not related to work engagement or career confidence.

There are also individual psychological factors which can contribute to assessments of the belongingness potential of social events, ranging from personal relationships to workplace situations. Rejection sensitivity is a “tendency to anxiously expect, readily perceive, and intensely react to rejection” (Downey and Feldman, 1996). It has been associated with negative early experiences and chronic exposure to stereotype and social identity threat (Aronson and McGlone, 2009) and is more common in minority groups (Maiolatesi et al., 2022). Where the anticipated rejection is linked to social identity, it can have the same effects as stereotype threat, with unnecessary responsivity to low threat, non-conflict events and consequent reduced performance (Derks et al., 2008). It can interact with organisational factors; high levels of rejection sensitivity, linked to reduced self-confidence and withdrawal from potential support mechanisms, have been shown among women in competitive, historically male institutions, or organisations with marked gender imbalances (London et al., 2012; Ahlqvist et al., 2013).

Belongingness has, therefore, been identified as a key factor to be considered in expectancy-value based approaches to occupational choices and engagement. Within the field of organisational psychology it has widened the scope of investigations into the reasons behind the existence and persistence of gender gaps (or, indeed, any kind of minority under-representation) in organisations.

It should be noted that the contemporary social psychological processes discussed above can be considered anticipatory in nature–overtly, in terms of expectancy-value theory, but also implicit in the “will I fit in and will I do well” aspects of belongingness theory or the anxious expectation of rejection sensitivity (Tellhed et al., 2017). So we have moved beyond individual reactions to incoming task demands to more outward-looking and future-focussed assessment of external social context and environment. This, of course, resonates well with the developments in social cognitive neuroscience detailed above.

## Summary

Here we have a useful conjunction of theoretical models in the study of career-based decisions, offering the possibility of a deeper understanding of apparently intransigent gender gaps. On the one hand, we have social cognitive neuroscience identifying the need to belong, or the avoidance of rejection, as a fundamental human drive. This is supported by the predictive coding approach to brain function, identifying the future-focussed, external rule-gathering nature of brain processes, equally applicable to social events as to sensory. On the other hand, we have the emerging emphasis in social psychology on context-based anticipation of social evaluation, identified by social cues and overt and covert messaging. Similarly, a need for positive social relationships and belongingness has been identified as a core behavioural drive.

Awareness of these new approaches should underpin any future interrogation of gender gaps, either for explanatory purposes or for the development of effective interventions. Theoretical or practical applications need to acknowledge the role of an organisational context in driving individual decision-making behaviour.

## Case study: The gender equality paradox–A new entry in an old playbook

*‘‘As we see it, the so-called gender equality paradox is a new entry in an old playbook of arguing that biological sex differences, not social inequalities, drive the gender disparities we see in areas such as STEM*.’’ ^10^

A paper published in 2018 reported the finding that women are more likely to be under-represented in the sciences in countries that have the highest levels of gender equality (Stoet and Geary, 2018). This would appear to be at odds with claims that a *lack* of gender equality had been behind the lack of women in science; reducing the gender equality gaps should, therefore, have resulted in *increasing* numbers of women in science (Williams and Ceci, 2015). Hence the paradox.

The authors had investigated STEM engagement between 2012 and 2015 in sixty-seven countries, reporting that fewer women than men were obtaining STEM degrees. They then linked this to the World Economic Forum’s Global Gender Gap Index (GGGI), reporting that in those countries where the gender gap was smallest (e.g., Finland, Norway, Sweden), the underrepresentation of women was highest.^11^ Performance scores on tests of scientific ability showed no female-male differences, eliminating any suggestion of cognitive deficit. [It should be noted that these “universal competence” data reveal (possibly inadvertently) a powerful argument against fundamental innate differences].

This paradox was interpreted in a variety of ways. One was that in the least gender equal countries, STEM jobs were better paid and so economic necessity drove the choices of both sexes. (In a similar vein to the above observation concerning innate competence, this appeal to economic drivers as determining career choice, demonstrates the potential role of external, social factors in the generation of gender gaps, rather than appeals to innate factors.). But in more gender equal countries, economic factors could take second place to the choice of a subject which “played to your strengths” and would be more likely to bring a sense of “efficacy and joy” (*sic*); life satisfaction could be given priority over economic necessity.

A newer form of biological determinism is evident in the narrative exploring these findings. Reference is made to “endogenous interests” (undefined) in determining career choice, suggesting that a choice between science and humanities is somehow internally determined: “We hypothesize that men are more likely than women to enter STEM careers because of endogenous interests……. Societal conditions can change the degree to which exogenous interests influence STEM careers (e.g., the possibilities of STEM careers to satisfy socio-economic needs). But *when there is an equal playing field* [own emphasis] and studying STEM is just as useful (balancing income and career satisfaction) as a degree in other areas, people are better able to pursue their interests and not simply their future economic needs” (Stoet and Geary, 2018).

So a 21st century explanation of gender gaps in science is still linked to a “natural” expression of some kind of individual innate differences. Possible contextual effects have been dismissed by reference to an “equal playing field,” inferring that narrow gender gaps are reliably associated with equality of opportunity or unbiased organisational cultures.

As we have seen, both contemporary social psychology and social cognitive neuroscience have emphasised the key role that social context will play in both engagement with, and persistence in, organisational culture, and demonstrated the brain- and behaviour-changing effects of bias in the workplace. So explorations of gender gaps in science should minimally include exploration of the effect of cultural factors in determining minority representation.

## The playing fields of science–Equal playing field or glass obstacle course?

“…*career pathways for women scientists and engineers are shaped by ideological and structural constraints, informal and formal biases, and active resistance or accommodation to them*” (De Welde and Laursen, 2011).

A primary challenge to the concept of equal playing fields could be to assess the extent to which any measure of gender equality or equity is associated with a reduction in “gatekeeping” stereotypes, as it has been demonstrated that these can exert a powerful influence on gender representation (Cheryan et al., 2015). With respect to claims about the paradoxical nature of science representation in countries with the lowest scores on the WEF’s GGGI, evidence shows that strong gender-science stereotypes still exist in these countries (Miller et al., 2015; Breda et al., 2020).

Stereotypical views can include whether or not women ‘‘belong’’ in science which, when aired in the public domain, could tip the balance against female engagement, given, as we have seen, the primary role of this factor in attributing value to vocational choices. In 2005, Larry Summers, then President of Harvard, caused an uproar by suggesting that the lack of high-achieving women in science and engineering was due to the ‘‘different availability of aptitude at the high end’’^12^; in 2017, James Damore, then a Google employee, voiced his opinion that in a memo that Google was wasting their time with diversity initiatives as “the distribution of preferences and abilities of men and women differ in part due to biological causes and …these differences may explain why we don’t see equal representation of women in tech and leadership’’^13^; in 2018, Alessandro Strumia, a theoretical physicist then working at European Council for Nuclear Research (CERN), made the case that male physicists were being unfairly discriminated against by a focus on reducing gender gaps in the face of evidence that “….the underrepresentation of women—reflects sound scientific evidence of gender differences in interests” (Banks, 2018). The undoubted problems with each of these statements have been well-documented elsewhere, but the very fact that, well into the 21st century, such views are still being widely aired cannot and should not be overlooked in any discussion of gender gaps.

Women within science also report a climate that can be contaminated by stereotypical attitudes. In physics, for example, a discipline characterised by marked and apparently intransigent gender disparities, a sense of belonging has been identified as having significant impact on performance and retention (Lewis et al., 2016). This was reportedly undermined by an (unsurprising) lack of role models and by the existence of negative ability stereotypes, for example, that women were more likely to make mistakes or show lower levels of competence. Women in physics have also reported that a form of stereotype-based sexist harassment, such as being exposed to sexist remarks about women’s lack of competence in physics or maths, or being ignored or put down or being treated differently, contributed (again) negatively to their sense of belonging (Aycock et al., 2019).

An extension of this female incompetence stereotype is the effect of implicit association of success in science with innate male “brilliance.” This refers to evidence of a belief in what is termed a form of “raw, innate talent” that is necessary to get you into the higher reaches of achievement in your particular field. In one study, academics from across 30 different academic disciplines in the US were asked to rate their agreements with statements such as “Being a top scholar of (x discipline) requires a special aptitude that just can’t be taught” (Leslie et al., 2015). This generated a “field-specific ability belief.” The percentage of female Ph.D. students in each subject were used to calculate gender gaps. In the sciences, the disciplines with the highest field-specific ability belief scores were engineering, computer science, physics and maths, which were also the subjects with the greatest gender gaps (Meyer et al., 2015). Academics in these fields were also more likely to agree with the statement “Even though it is not politically correct to say it, men are more often suited than women to do high-level work in (x discipline).” This “belief in brilliance” has also been shown to be associated with a lower sense of belonging in female STEM students (Deiglmayr et al., 2019). A very recent survey has reported that the stereotype associating talent with men showed that this is a world-wide phenomenon, as measured by a survey of more than half a million students in 72 countries (Napp and Breda, 2022).

With respect to the value that a career in science might offer, scrutiny of career progression and reward systems can provide clues to prospective entrants. There are many ways in which success is measured in science. This can include publications, citations, grant income. In all of these spheres, there is clear evidence of bias (Llorens et al., 2021).

With respect to publications, surveys of editorships and membership of editorial boards provide clear evidence of gender imbalance (Lundine et al., 2018). As the entry point into this Key Performance Indicator of academic success, such bias can determine what gets published and who gets published… a “powerful influencing factor in the landscape of scholarship” (Lin and Li, 2023). Even where the representation of women in a scientific discipline is proportionally high, it is clear that they do not proportionally achieve the positions of power offered by various editorship roles (Lin and Li, 2023).

Several studies have noted a marked gender imbalance in authorship, which could not be put down to quality or submission rates, particularly in more prestigious journals (Bendels et al., 2018; Holman et al., 2018).^14^ Once published, a measure of a publication’s quality is how often it gets cited. Again, there is evidence that papers with female key authors are cited less frequently (Dworkin et al., 2020), possibly related to gender differences in self-citation (Chawla, 2016). Studies of success in grant funding have uncovered gender disparities, with men receiving higher evaluations in the ‘quality of applicant ‘categories despite no gender differences in “quality of proposal” evaluations (Van der Lee and Naomi, 2015; Witteman et al., 2019).

On a variety of measures then, it would appear that science sends out many signals about who may or may not belong–there would not yet appear to be an equal playing field. As we have seen, lack of a sense of belonging is a powerful driving force in informing human decision making. Thus, the choice of many competent (and allegedly empowered) individuals to seek their futures elsewhere would appear to be less paradoxical than has been asserted. The absence of women from science can indeed be cast as a brain-based problem, but not one to do with internally determined, individual cognitive capacity but rather in terms of dynamic interactions with external factors, importantly, external factors which can be changed appropriately. The link between brains and their world is not a one-way street.

Explanations for the Gender Equality Paradox have focussed on allegedly unconstrained choices *not* to follow a career in science made by competent females, leading to apparently intransigent gender gaps. Reference is made to science as a “levelled playing field,” with the inference that gendered expectations around science and scientists, and historical biases in organisational processes such as recruitment and retention, have broadly been eliminated. However, closer examination of the cultural context of science and scientific organisations demonstrates that this is not the case. There is evidence of lingering stereotypes of science as a male domain, and of structural biases against women within systems supposedly supporting career progression and reward. These are the very organisational factors that both contemporary social cognitive neuroscience and social psychology have identified as key to establishing negative expectations and driving avoidance behaviour within under-represented groups, in this case, females.

Networks in the social brain can be activated by the very anticipation of negative social experiences, and will elicit behavioural inhibition and avoidance behaviour. Expectations would be established by externally-focussed, rule-gathering predictive coding activity within the brain. If overt or covert messaging within any organisation flags that an individual would be in the minority, might not be viewed as competent and/or would be unlikely to progress, these could be perceived as “rules” that should drive future behaviour, with the end goal of supporting a drive to belong and ensuring the maintenance of self-esteem. In social psychology terms, the potential lack of “belongingness,” signalled by such messaging, would serve as a powerfully negative value in any assessment of psychological or social costs or benefits of joining such an organisation.

## Conclusion: Embedded beings and embedded brains

The intransigence of gender gaps are a problem for society and social progress as well as an issue of social justice. Explanations (and justifications) for such gaps have a long and sometimes chequered history. Traditional essentialist explanations focussed on issues of individual competence, locating the problem in the innate, fixed and invariant consequences of pre-determined biological factors. Early social explanations still focussed on individuals, their life-choice decisions shaped by social forces such as stereotyping and role expectations.

This paper suggests that there has been a refocussing in both approaches, on inter-personal factors such as group membership or belongingness as a fundamental need, and on the role of environmental clues and external messaging in the anticipation and assessment of positive or negative social experiences. The proactive, future-focussed characterisation of human brain function and the identification of powerful inhibitory forces in socio-affective brain circuitry, activated by negative social experiences such as social rejection, dovetails well with the incorporation of anticipated or experienced belongingness into the expectancy-value framework evident in contemporary social psychology research.

As yet, the developments in social neuroscience are less evident in the field of organisational research, although their relevance to gender gaps has been raised.^15^ But the evidence of social inclusion as a fundamental human need fits well with belongingness research, and offers confirmatory evidence of inferred internal processes such as rejection sensitivity or social identity threat. Working together, these two approaches could offer a fruitful way forward toward identifying and dismantling the organisational barriers which still deter engagement and discourage retention, and perpetuate gender gaps. And, further, the social cognitive neuroscience model outlined above could well be applied to any underrepresented groups in any organisations. The importance of a sense of inclusion, of belongingness, is equally true for ethnic minorities, for people with disabilities, for the neurodiverse and the gender diverse. We need to address the issue of place as well as people, of inclusion as well as diversity, and genuinely ensure an equal playing field for all.

## Author contributions

The author confirms being the sole contributor of this work and has approved it for publication.

## Footnotes

1
https://www.weforum.org/reports/global-gender-gap-report-2022/

2
https://execpipeline.com/wp-content/uploads/2021/07/Women-Count-2021-Report.pdf

3
https://www.catalyst.org/research/women-ceos-of-the-sp-500/

4
https://www.wes.org.uk/content/wesstatistics

5
https://www.womentech.net/en-gb/women-technology-statistics

6
https://www3.weforum.org/docs/WEF_GGGR_2020.pdf

7
https://royalsociety.org/topics-policy/projects/dynamics-of-data-science/

8 President’s Council of Advisors on Science and Technology. Engage to excel: producing one million additional college graduates with degrees in science, technology, engineering, and mathematics (Executive Office of the President of the United States, 2012).

9
https://www.fawcettsociety.org.uk/unlimited-potential-the-final-report-of-the-commission-on-gender-stereotypes-in-early-childhood

10
https://uk01.l.antigena.com/l/ZUSAwcvQL66zA33cFeXxU9dZ9YwiPG_cUBb-1c8Ztx1rlSMEIjnoHL1KixCZctdPkB4gJMs1BdQvANfxtCcXywttxgNouuteTTxrIFaxsyk8g2Mc48otaEyXlmu4A_4-mrUm6K5QzrqDLGKIsf-d-SlGdGu4nihs_AqZm3IdLojS6PDstlVDh4ox6B

11 There is some debate about how the authors generated the data that came up with this conclusion. An unorthodox way of measuring science choice mixed with a somewhat personalised tweak of the GGGI suggests that their correlational data may not anyway be telling such a clear-cut story. Nevertheless, the essentialist interpretation in their discussion is an interesting example of a newly emerging narrative round sex differences and gender gaps.

12
https://www.thecrimson.com/article/2005/2/18/full-transcript-president-summers-remarks-at/

13
https://www.documentcloud.org/documents/3914586-Googles-Ideological-Echo-Chamber.html

14
https://www.rsc.org/new-perspectives/talent/gender-bias-in-publishing/

15 A Gendered World Makes a Gendered Brain https://www.youtube.com/watch?v=2s1hrHppl5E.
